# Stress Characteristics and Their Associations With Pain Intensity: An Ecological Momentary Assessment Study

**DOI:** 10.1002/ejp.70240

**Published:** 2026-02-25

**Authors:** Karen Ghoussoub, Mael Gagnon‐Mailhot, Élise Develay, Pierre Rainville, Sonia Lupien, Lise Dassieu, Mathieu Roy, Étienne Vachon‐Presseau, M. Gabrielle Pagé

**Affiliations:** ^1^ Centre de Recherche du Centre Hospitalier de l'Université de Montréal (CRCHUM) Montréal Canada; ^2^ Department of Psychology, Faculty of Arts and Sciences Université de Montréal Montréal Canada; ^3^ Department of Stomatology, Faculty of Dental Medicine Université de Montréal Montréal Canada; ^4^ Centre de Recherche de l'Institut Universitaire de Gériatrie de Montréal (CRIUGM) Montréal Canada; ^5^ Department of Psychiatry and Addictology Faculty of Medicine, Université de Montréal Montréal Canada; ^6^ Centre de Recherche du CIUSSS‐du‐Nord‐de‐l'île‐de‐Montréal Montréal Canada; ^7^ School of Social Work Université du Québec à Montréal Montréal Canada; ^8^ Department of Psychology Faculty of Science, McGill University Montréal Canada; ^9^ Faculty of Dentistry McGill University Montréal Canada; ^10^ Department of Anesthesiology and Pain Medicine, Faculty of Medicine Université de Montréal Montréal Canada

## Abstract

**Background:**

This study aimed to understand the associations between stress intensity, characteristics that trigger a physiological stress responses (Sense of lack of control, social evaluative Threat, Unpredictability, Novelty; STUN) and the experience of momentary pain intensity and its variability among adults living with chronic low back pain.

**Methods:**

Participants (*n* = 181) completed electronic diaries three times daily for 7 days that measured pain and stress intensity (Numeric Rating Scale‐11) and the extent to which stressors were attributable to the STUN characteristics. Data was analysed using a Mixed‐Effects Location Scale Model.

**Results:**

A 1‐point increase in between‐subject (BS) stress intensity was associated with a 0.70 point (*p* < 0.001) increase in pain intensity. For every 1‐point increase in BS and within‐subject (WS) perceived lack of control, pain intensity increased respectively by 0.128 and 0.040 point (*p* < 0.01). A 1‐point increase in WS novelty was associated with a 0.05‐point decrease in pain intensity (*p* < 0.001). There was more homogeneity in pain scores across participants at higher levels of WS stress intensity, WS perceived lack of control (β = −0.032) and BS perceived novelty (β = −0.243) (*p* < 0.05). Participants' own pain intensity ratings were not more or less consistent or erratic as a function of their stress levels.

**Conclusions:**

Stress intensity is associated with pain intensity and with BS variance in pain ratings. STUN characteristics were inconsistently associated with pain variance, but lack of perceived control and novelty might be associated with pain intensity. Future research should clarify the role of STUN characteristics, above stress intensity, in pain intensity and pain variability.

**Significance Statement:**

This study advances understanding of stress–pain dynamics by applying the integrative STUN framework to chronic low back pain. Findings reveal that perceived lack of control amplifies pain, whereas novel experiences reduce it. By identifying specific stress characteristics influencing pain variability, this work highlights innovative intervention targets, emphasising the potential of fostering novelty and re‐evaluating control perceptions in chronic pain management.

## 1 Background

Low back pain (LBP) is one of the most prevalent types of chronic pain (Henschke et al. 2008; Hoy et al. 2012) and one of the leading global causes of disability (Cieza et al. 2021). LBP is multidimensional and requires a biopsychosocial framework to understand and manage it (Gatchel and Turk 1996). Stress, being at the intersection of these biological, psychological and social components, is an important contributor to chronic LBP (cLBP) (Wippert and Wiebking 2018). Experimental and clinical evidence indicate that stress can have paradoxical effects on pain, namely hypoalgesia and hyperalgesia (Olango and Finn 2014; Vachon‐Presseau et al. 2013). Acute or controllable stressors can temporarily suppress pain perception by activating descending inhibitory pathways that involve endogenous opioids and endocannabinoids. Prolonged, uncontrollable or repeated stress exposure can dysregulate the stress systems and facilitate nociceptive processes leading to increased pain sensitivity (Ferdousi and Finn 2018; Jennings et al. 2014). These dynamic interacting systems are relevant to understand chronic pain conditions like cLBP, as persistent exposure to stress may shift the balance toward hyperalgesic states and pain maintenance.

In this context, it is important to understand the four situational characteristics that lead to a physiological stress response (Dickerson and Kemeny 2004; Mason 1968): Sense of low control (S), social evaluative Threat (T), Unpredictability (U) and Novelty (N) [STUN] (Lupien et al. 2013). These characteristics have been shown to activate the hypothalamic–pituitary–adrenal (HPA) axis, which, when the brain detects a threat, leads to a cascade of reactions ultimately leading to cortisol secretion from the adrenal glands (Dickerson and Kemeny 2004; Mason 1968).

Observational and experimental studies showed heterogeneous results about the impact of some of the STUN characteristics on the pain experience (Ghoussoub et al. 2024). A qualitative study showed that the STUN model is relevant to explain how stress influences various dimensions of the pain experience (Pagé et al. 2021a).

Examining how stress and the STUN characteristics are associated with pain intensity is important but provides an incomplete understanding of the pain experience. Pain is increasingly recognised as a dynamic state (Schneider et al. 2012; Stone, Broderick, et al. 2021; Winger et al. 2019). Increased variability in pain experiences is associated with increased avoidance behaviours, disability, depressive symptoms, lower quality of life, less effective coping strategies and greater pain severity (Bartley et al. 2018; Rogers et al. 2025; Wesolowicz et al. 2021; Zakoscielna and Parmelee 2013) and as such it is important to understand factors that influence this variability.

**FIGURE 1:**
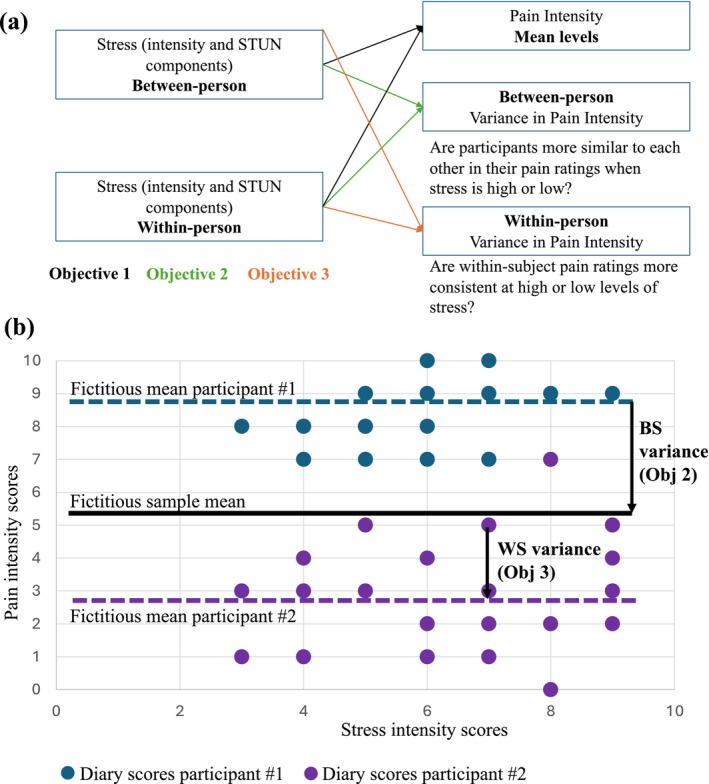
Panel A illustrates the study's objectives. Panel B provides an example, using two fictitious cases, of what objectives 2 and 3 explore. BS variance: The extent to which an individual deviates from the group mean response represents the BS variance. Therefore, if all participants' dotted lines are close together, then there is little subject heterogeneity, and if they are far apart, then there is great subject heterogeneity. WS variance: The extent to which an individual's dots are close or far away from their own mean (dotted line).

For example, among adults with rheumatoid arthritis, mood, but not anxiety, was associated with pain variability (Schneider et al. 2012). It is possible that the STUN model, by highlighting the extent to which pain fluctuations can be unpredictable or uncontrollable, for example, can influence pain perception and contribute to increased overall pain intensity but also increased variability in pain across and within participants. This study aimed to understand the associations between stress intensity, STUN characteristics, and the experience of momentary pain intensity and its variability among individuals living with cLBP (see Figure 1). More specifically, this study examined whether:

- Higher levels of between‐subject (BS) and within‐subject (WS) stress levels (intensity and STUN components) are associated with overall greater pain intensity scores;
- Higher levels of BS and WS stress levels (intensity and STUN components) are associated with increased BS variance in pain intensity, that is, whether participants' pain scores are more or less similar to each other; and
- Higher levels of BS and WS stress levels (intensity and STUN components) are associated with increased WS variance in pain intensity, that is, whether a participant's own pain ratings are more or less consistent.

## 2 Methods

This quantitative study is part of a larger prospective longitudinal mixed methods project. Participants completed baseline and follow‐up self‐reported measures and a 1‐week electronic diary in between; subgroups of participants also provided saliva samples (to measure cortisol levels) and/or participated in a qualitative interview. The present study focused on the ecological momentary assessment period. The study was approved by the research ethics board of the *Centre hospitalier de l'Université de Montréal* (20.347‐YP). The Adapted STROBE Checklist for Reporting EMA Studies (CREMAS) was followed in this study (Liao et al. 2016).

### 2.1 Participants

Eligible participants were adults living with cLBP (> 3 months duration) reporting moderate to severe pain intensity (Numeric Rating Scale score of average pain intensity ≥ 4/10), fluent in written and spoken French and/or English and with access to the Internet. Exclusion criteria included cognitive/physical impairments that made it impossible to complete the diaries, and reporting back pain due to cancer or cancer‐related treatments. Chronic cancer‐related pain has a different, possibly life‐threatening meaning (Farquhar‐Smith 2016), which represents a different type of threat/stress compared to the experience of individuals living with non‐cancerous chronic pain.

### 2.2 Recruitment

Participants were mostly recruited between March 2021 and May 2022 from the Quebec Low Back Pain Study (https://backpainconsortium.ca) (QLBPS), an initiative of the Quebec Low Back Pain Consortium within the Quebec Pain Research Network that includes over 7000 individuals living with LBP; more than 80% of these participants have consented to be contacted to participate in various sub‐studies (Pagé et al. 2020). Individuals in the QLBPS are recruited primarily via (a) social media, but also via (b) conventional media and advertisements, (c) email list servers, (d) advertisements with patient or professional organisations, (e) unions of workers and (f) advertisements in medical settings (Pagé et al. 2020).

To increase the representativeness of the study sample, we also recruited participants from physiotherapy, physiatry and medical clinics in the Greater Montréal area using flyers.

Potential participants were contacted by phone by a research assistant who determined eligibility and obtained verbal consent by phone.

### 2.3 Procedure

**FIGURE 2:**
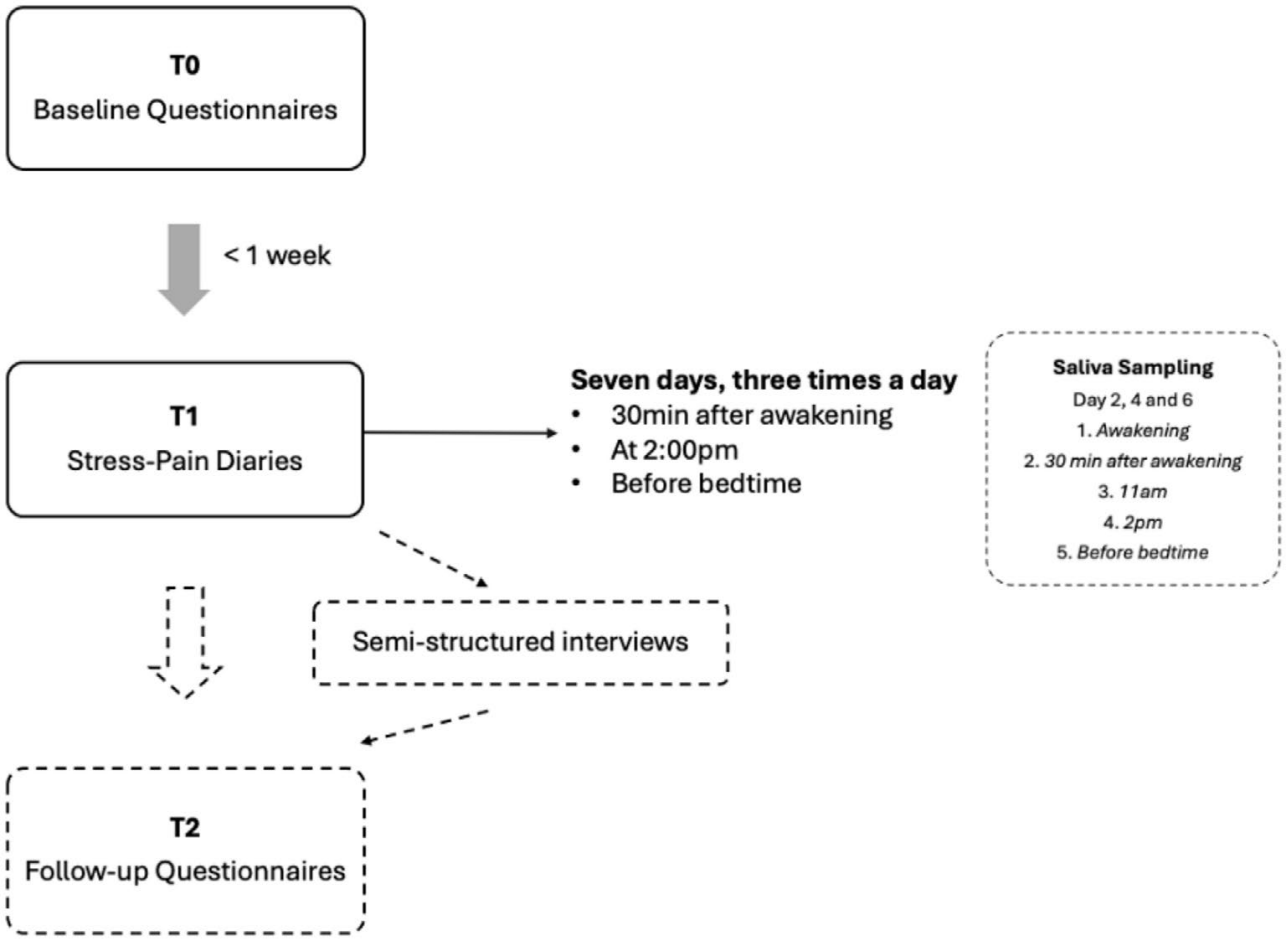
Stress‐pain study procedure. T0 and T1 are included in the current study.

Eligible participants who gave verbal consent were emailed a link to the electronic consent form via the Research Electronic Data Capture (REDCap) platform (Harris et al. 2009). After providing written consent, participants had 1 week to complete the baseline questionnaires. Following completion, participants downloaded the MyCap application (https://www.projectmycap.org), an external module of REDCap, on their device (or a pre‐programmed iPod touch borrowed from the research team) and were accompanied by a research assistant to resolve any technological issues. Participants completed a stress‐pain diary for 7 days, three times a day (30 min after awakening, at 2:00 p.m. and before bedtime) (see Figure 2). These specific number and timing of daily assessments were chosen to strike a balance between optimal data timepoints and minimise participant fatigue, capture circadian fluctuations in pain intensity, and facilitate saliva sample collection (saliva samples are beyond the score of this study). Given that missing data increases with time in EMA studies, the data collection was limited to 7 days (Ono et al. 2019). Each diary contained 11 questions about their current state (*right now*) and took < 2 min to complete. Participants were instructed to set reminder alarms on their device to complete the diaries on time. A research assistant contacted them after 3 days to optimise diary completion rates. A subgroup of participants also completed saliva samples five times daily during days two, four and six of the diary week. Some participants also took part in an individual semi‐structured interview to further explore the relationship between stress and pain. Finally, participants completed follow‐up questionnaires 2 months later. The saliva samples, qualitative interviews and follow‐up questionnaires are beyond the scope of this study. Participants received a financial compensation for participating in the study.

### 2.4 Measures

Only measures used for the present analysis are described below (see File S1 for the complete list).

#### 2.4.1 Measures—Baseline Questionnaire

##### 2.4.1.1 Canadian Minimum Dataset for Chronic Low Back Pain Research

This questionnaire is a cross‐cultural adaptation of the National Institutes of Health Task Force Research Standards on LBP (Lacasse et al. 2017). It measures various aspects of back pain, including duration, intensity (NRS‐11: 0 = no pain and 10 = worst pain imaginable), presence of sciatica, previous treatments and work absenteeism. This questionnaire also uses items from the Patient‐Reported Outcomes Measurement Information System (PROMIS) and the STarT Back Screening Tool to assess pain interference, emotional distress (depressive symptoms) and sleep (Bruyère et al. 2012; Hill et al. 2008). These scales have been validated in several populations, including individuals living with chronic musculoskeletal pain (Deyo et al. 2015; Deyo et al. 2016). Finally, it documents the participants' history of substance use, smoking status, height, weight, age, gender, ethnicity, education, occupational status and current litigation.

#### 2.4.2 Ecological Momentary Assessment Questions

The electronic diary (see File S2) included questions based on self‐administered numerical rating scales (NRS‐11) to measure current perception of pain intensity (0 = no pain at all, 10 = worst pain imaginable) and stress intensity (0 = not stressed at all, 10 = most stressed imaginable). Pain intensity was chosen over pain interference as it more closely aligns with the literature examining the association between pain intensity and stress (hyperalgesic and hypoalgesic effects), which is the focus of the present study. Visual analogue scales (VAS‐100) (Haefeli and Elfering 2006) measured the degree to which stress was associated with each of the STUN characteristics (0% = not at all; 100% = completely):

- How much control do you feel you have over the stressful situation(s) you are currently facing? This item was reverse‐scored in the analysis, so that higher scores indicate a greater sense of low control.
- How novel (new) are the stressful situation(s) you are currently facing?
- How predictable/unpredictable are the stressful situation(s) you are currently facing?
- How threatening to your personality are the stressful situation(s) you are currently facing?

To ease the interpretation of the model, the STUN variables were brought back to a 0–10 scale. Given that these questions were created for the purpose of this study and are not validated, results generated are exploratory in nature. The diary also included questions about mood, fatigue and stress attributions (pain, pandemic and other). The NRS for pain intensity has been shown to have good reliability, validity and sensitivity to change (Jensen and Karoly 2001). The VAS has been shown to have good validity and discriminative sensitivity when measuring stress (Lesage et al. 2012). The electronic diaries were pilot tested before initiating the present study (Pagé et al. 2021b).

### 2.5 Data Analysis

Descriptive analyses were conducted in IBM‐SPSS v.27 while the main analyses were conducted in MixWILD version 2.0 (Dzubur et al. 2020). Participants who completed under 50% of electronic diaries (i.e., < 10 electronic diaries) were excluded from the main analyses due to a low compliance rate (see Section 2.5.2). For the first daily diary (30 min after awakening), diaries completed before noon were eligible given the variable awakening time across participants. For the second diary (2:00 p.m.), a time frame of 2 h before or after that time was accepted. For evening diaries (before bedtime), diaries completed up to 4:00 a.m. were eligible to accommodate those going to bed later (when their other diary entry points were also shifted consequently) but ensuring no overlap between the three diary times. The main analyses only included diary entries where the stress intensity score was > 0, to test the effects of the STUN characteristics (i.e., the extent to which their reported stress was associated with each of the STUN characteristics) (see Section 2.5.2).

A Mixed‐Effects Location Scale Model (MELS) is an extension of a multilevel model that includes a location (random subject intercept that corresponds to a subject's mean) and a scale (random subject scale that corresponds to the subject's variance) (Hedeker et al. 2012) and was used to answer the study objectives. The MELS model decomposed the pain intensity outcome into its mean (mean value of pain submodel), BS variance (BS variance in pain ratings submodel), and WS variance (WS variance in pain ratings submodel). The mean value of pain submodel examined whether stress levels were associated with overall pain intensity (location; Objective 1). The BS variance in pain ratings submodel (scale; Objective 2) examined whether stress levels were associated with the extent to which individuals were similar/dissimilar in their overall pain scores. The WS variance in pain ratings submodel (scale; Objective 3) examined whether stress was associated with how consistent/erratic an individual was in their pain scores across the diary period. In each model, the effects of stress were decomposed into their BS (differences in stress ratings across participants) and WS (changes in pain ratings within a participant) components.

No data imputation strategies were used given that all diary variables had < 5% of missing data.

#### 2.5.1 Model Specification

The specified MELS model was built in three steps, starting with random subject intercepts (location effects), and then adding random scale effects to capture individual differences in the variance of pain intensity ratings across time. The model included five time‐varying continuous variables (stress intensity (0–10) and the STUN characteristics (0–10)) simultaneously entered in the model, and desegregated into their BS and WS effects.

#### 2.5.2 Sensitivity Analyses

The main analysis included participants with 10 or more completed diaries, and included only diaries with stress intensity scores > 0. The following sensitivity analyses were conducted and presented in the results when significant differences were found with the main analysis:

- A sensitivity analysis using the entire sample (*n* = 217); thus, including participants who completed at least one diary.
- A sensitivity analysis using participants with 10 or more completed diaries and including all stress intensity scores (including those equal to 0).
- A sensitivity analysis using only participants with 10 or more completed diaries, who have at least on average once per day (so at least seven diary entries) with a stress intensity score > 0.
- A sensitivity analysis using only participants with 10 or more completed diaries, who have at least on average once per day (so at least seven diary entries) a stress intensity score ≥ 4.

#### 2.5.3 Sample Size

To examine the location submodel, a total sample of 147 participants would be required to detect a small effect size (0.10), with 80% power with six variables of interest (Abramowitz and Stegun 1964; Cohen 1988; Raudenbush and Bryk 2002; Soper 2019). A total sample of 181 participants was included in this study. This power analysis only applies to objective 1 of the study and does not consider all of the complexity of the MELS models.

## 3 Results

**FIGURE 3:**
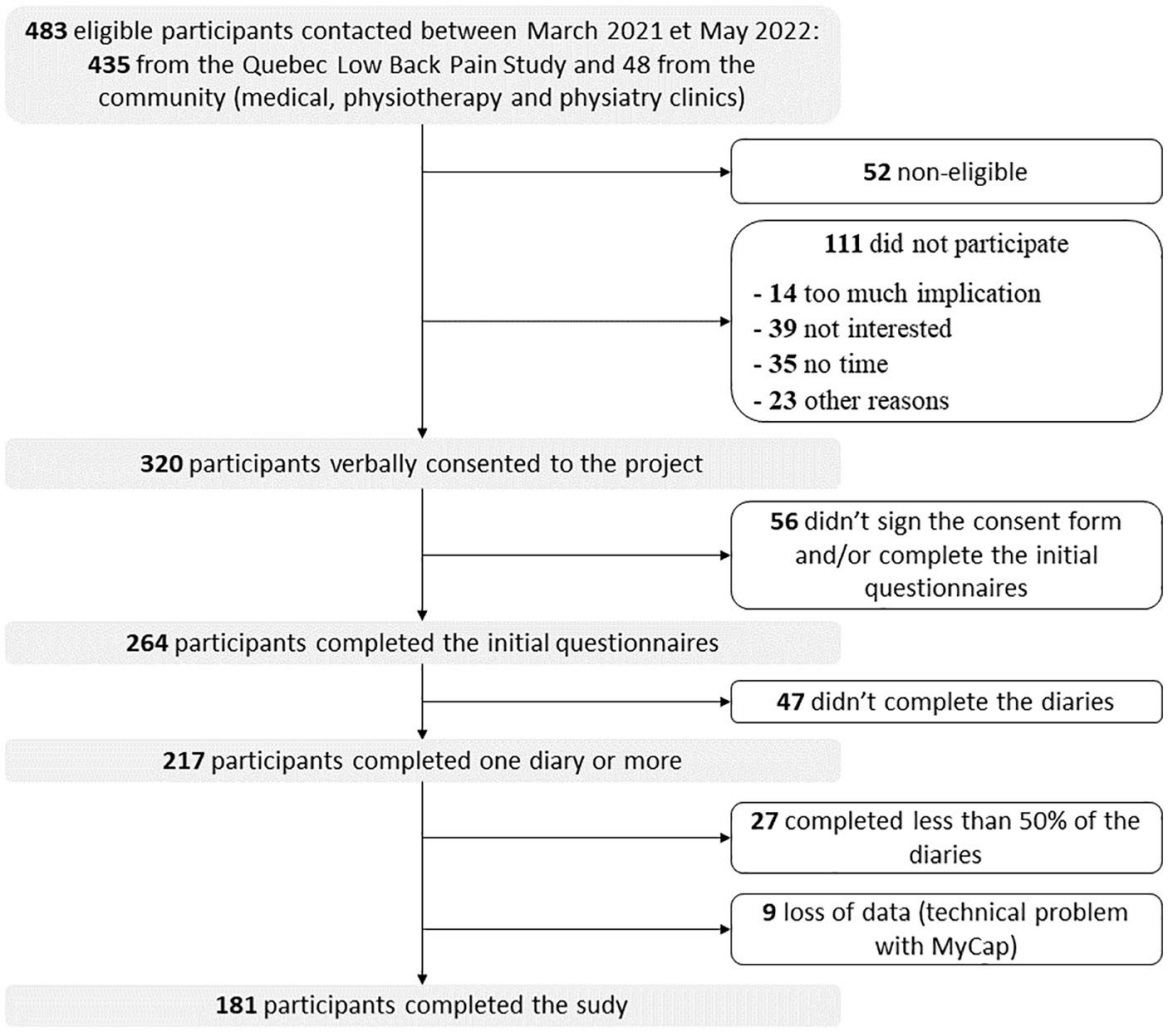
Study flow chart.

**FIGURE 4:**
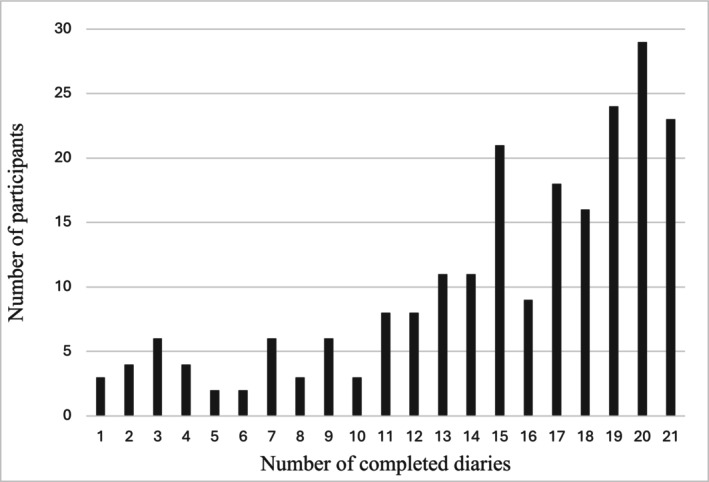
Completion rates for electronic diaries. The completion rate of electronic stress‐pain diaries of participants who completed one or more diaries (*n* = 217).

In total, 483 people were contacted for this project, 276 signed the written consent, 264 completed the initial questionnaires, and 217 participants completed at least one electronic diary entry. Due to a technical problem on the server, data from nine participants were lost (see Figure 3). Electronic diary completion rates for participants who completed one or more diaries are shown in Figure 4. A total of 181 individuals completed 10 or more diaries and were included in the main analyses. 90% of the individuals were recruited from the Quebec Low Back Pain Study, and the remaining 10% from clinics in the Greater Montreal.

### 3.1 Sociodemographic, Stress and Pain Characteristics

The baseline sociodemographic, stress and pain characteristics of the participants are reported in Table 1. Participants were on average 49.5 ± 12.5 (mean ± standard deviation) years old. More than half of the sample was composed of women (58.3%). Most participants identified as White (83.1%) and reported having completed a college or university degree (74.5%). More than half of the participants (55%) were working. Most participants (74.6%) had been living with pain for more than 5 years. The average pain intensity at baseline was 6.0 ± 1.7 (on a scale of 0–10). According to the PROMIS T‐Scores computed from the self‐administered questionnaires, participants reported clinically (Kroenke et al. 2020; Rothrock et al. 2020) mild depressive symptoms (57.3 ± 9.0), anxiety (56.8 ± 9.7) and sleep disturbances (55.4 ± 7.7) and moderate pain interference levels (61.2 ± 7.4) (Rothrock et al. 2020).

**TABLE 1:** Sociodemographic, stress and pain characteristics of the study participants measured at baseline.

Characteristics mean ± SD or *N* (%)	Participants who completed 10 diaries or more (*n* = 181)	Total (*n* = 217)
Sociodemographic characteristics		
Age (years)	49.5 ± 12.5	48.7 ± 12.4
Sex		
Woman	105 (58.3)	125 (57.9)
Man	75 (41.7)	91 (42.1)
Missing	1	1
Gender identity		
Woman	106 (58.9)	125 (58.1)
Man	74 (41.1)	90 (41.9)
Missing	1	2
Ethnicity		
White	148 (83.1)	180 (84.1)
First Nations	2 (1.1)	2 (0.9)
Métis	3 (1.7)	3 (1.4)
Member of a visible minority	21 (11.8)	25 (11.7)
Member of an ethnic minority	4 (2.2)	4 (1.9)
Missing	3	3
Education		
High school or less	46 (25.5)	55 (25.4)
Collegial	57 (31.7)	73 (33.8)
University	77 (42.8)	88 (40.7)
Missing	1	1
Work status		
Worker	99 (55.0)	124 (57.4)
Disability (temporary or permanent)	32 (17.8)	36 (16.7)
Retired	26 (14.4)	30 (13.9)
Other	23 (12.8)	26 (12.0)
Missing	1	1
Current litigation		
Yes	7 (4.0)	8 (3.8)
No	169 (96.0)	204 (96.2)
Missing	5	5
Pain and stress characteristics		
Pain duration		
> 3–11 months	5 (2.8)	6 (2.8)
1–5 years	41 (22.7)	57 (26.3)
> 5 years	135 (74.6)	154 (71.0)
Radiating pain		
Yes	110 (64.7)	132 (65.7)
No	60 (35.3)	69 (34.3)
Missing	11	16
Pain and stress characteristics		
Mean pain intensity during the past 7 days (0–10)	6.0 ± 1.7	6.1 ± 1.8
PROMIS—Interference	61.2 ± 7.4	61.1 ± 7.1
PROMIS—Depressive symptoms	57.3 ± 9.0	57.5 ± 8.9
PROMIS—Anxiety	56.8 ± 9.7	56.7 ± 9.5
PROMIS—Sleep disturbance	55.4 ± 7.7	55.6 ± 7.7

Abbreviations: PROMIS, Patient‐Reported Outcomes Measurement Information System; SD, standard deviation.

The mean pain and stress intensities during the diary period were 4.2 ± 2.2 and 2.8 ± 2.4/10, respectively (see Table 2). On average, participants reported that their stress was characterised by low perceived control (5.5 ± 3.3) and unpredictability (4.2 ± 3.2) and to a lesser extent novelty (2.9 ± 3.1) and social‐evaluative threat (2.2 ± 2.9).

**TABLE 2:** Stress and pain characteristics measured during the electronic diaries' week.

Characteristics	Participants who completed 10 diaries or more (*n* = 181)		Total (*n* = 217)	
	Mean ± SD	ICC	Mean ± SD	ICC
Pain characteristics				
Pain intensity (0–10)	4.2 ± 2.2	0.557	4.3 ± 1.7	0.340
Stress characteristics				
Stress intensity (0–10)	2.8 ± 2.4	0.503	2.9 ± 2.0	0.341
Sense of low control (0–10)^a^	5.5 ± 3.3	0.473	5.6 ± 2.3	0.342
Threat to ego (0–10)^a^	2.2 ± 2.9	0.667	2.3 ± 2.5	0.342
Unpredictability (0–10)^a^	4.2 ± 3.2	0.430	4.3 ± 2.3	0.342
Novelty (0–10)^a^	2.9 ± 3.1	0.461	2.9 ± 2.3	0.340

Abbreviations: ICC, intra‐class correlation coefficient; SD, standard deviation.

^a^ A higher score indicates a greater presence of the STUN characteristics.

### 3.2 Daily Associations Between Stress and Pain Intensity

**FIGURE 5:**
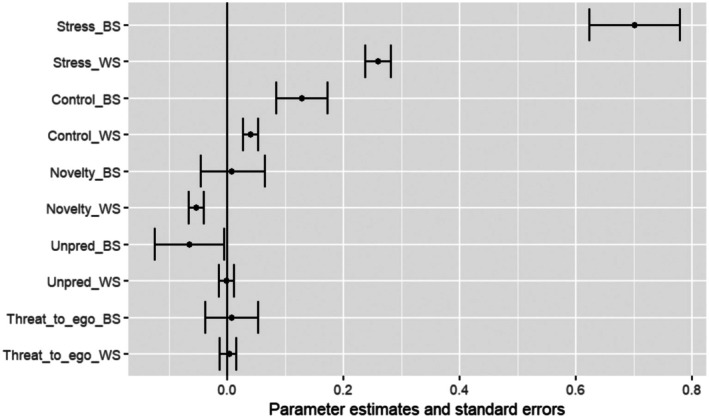
Regression coefficients and standard errors of the mean value of pain submodel of the MELS model.

**FIGURE 6:**
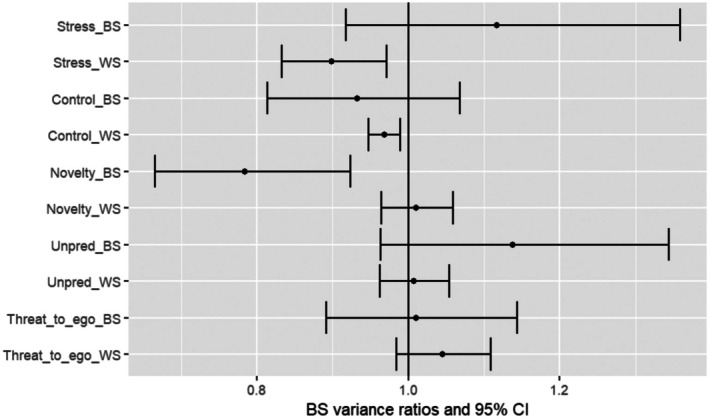
Between subject variance ratios in pain ratings and confidence intervals of the between subject variance in the pain ratings submodel of the MELS model.

Results of the MELS model are reported in Table 3, Figures 5 and 6.

**TABLE 3:** Mixed‐effects location scale models examining the associations between stress characteristics and pain intensity.

Variable and parameters	Estimate	SE	*p*	Ratio	95% CI	
					Lower	Upper
Mean model						
Intercept	1.474	0.411	0.005*	—	—	—
Stress (BS)	0.701	0.077	< 0.001**	—	—	—
Stress (WS)	0.259	0.022	< 0.001**	—	—	—
Control (BS)	0.128	0.044	0.004*	—	—	—
Control (WS)	0.040	0.013	0.003*	—	—	—
Novelty (BS)	0.008	0.053	0.886	—	—	—
Novelty (WS)	−0.053	0.013	< 0.001**	—	—	—
Unpredictability (BS)	−0.065	0.060	0.279	—	—	—
Unpredictability (WS)	−0.001	0.013	0.921	—	—	—
Threat to ego (BS)	0.007	0.045	0.871	—	—	—
Threat to ego (WS)	0.003	0.017	0.835	—	—	—
Between‐subject variance in pain ratings						
Intercept	0.524	0.554	0.343	1.689	0.571	4.999
Stress (BS)	0.110	0.100	0.270	1.117	0.918	1.359
Stress (WS)	−0.106	0.039	0.007*	0.899	0.833	0.971
Control (BS)	−0.070	0.070	0.314	0.933	0.814	1.068
Control (WS)	−0.032	0.011	0.003*	0.968	0.948	0.989
Novelty (BS)	−0.243	0.084	0.004*	0.784	0.665	0.924
Novelty (WS)	0.010	0.024	0.664	1.010	0.964	1.059
Unpredictability (BS)	0.129	0.085	0.130	1.138	0.963	1.344
Unpredictability (WS)	0.007	0.023	0.763	1.007	0.962	1.054
Threat to ego (BS)	0.010	0.063	0.872	1.010	0.892	1.144
Threat to ego (WS)	0.044	0.030	0.151	1.045	0.984	1.109
Within‐subject variance in pain ratings						
Intercept	0.641	0.231	0.005*	1.899	1.208	2.985
Stress (BS)	0.067	0.044	0.124	1.070	0.982	1.166
Stress (WS)	0.036	0.025	0.146	1.037	0.988	1.088
Control (BS)	−0.029	0.027	0.291	0.971	0.921	1.025
Control (WS)	−0.003	0.015	0.856	0.997	0.969	1.026
Novelty (BS)	−0.034	0.035	0.313	0.966	0.902	1.033
Novelty (WS)	−0.003	0.016	0.875	0.997	0.966	1.030
Unpredictability (BS)	−0.009	0.039	0.823	0.991	0.918	1.070
Unpredictability (WS)	−0.022	0.016	0.156	0.978	0.949	1.008
Threat to ego (BS)	−0.030	0.026	0.244	0.971	0.923	1.021
Threat to ego (WS)	−0.016	0.020	0.437	0.984	0.946	1.024
Random scale standard deviation						
Standard deviation	0.579	0.049	< 0.001**	1.785	1.622	1.963
Random location (mean) effect on WS variance						
Location effect	0.045	0.065	0.495	1.046	0.920	1.188

*Note:* *p < 0.05; **p < 0.001.

Abbreviations: BS, between‐subject; WS, within‐subject.

#### 3.2.1 Effects of Stress on Mean Pain Intensity Across Diaries (Location)

Both BS and WS stress intensity levels were associated with overall pain intensity. Specifically, for each 1‐point increase in participants' overall stress intensity (BS), their overall pain intensity increased by 0.70 points (*p* < 0.001). In addition, for every 1‐point increase in their stress intensity score compared to their own mean (WS), participants' pain intensity increased by 0.26 points (*p* < 0.001).

Beyond the overall significant positive association between stress and pain intensity, two STUN characteristics displayed significant effects. For every 1‐point increase in perceived lack of control across participants during the diary period (BS), pain intensity was increased by 0.128 points (*p* < 0.01). For every 1‐point increase in perceived lack of control compared to their own mean (WS), participants' pain intensity increased by 0.040 points (*p* < 0.01). Higher WS novelty was associated with decreased pain intensity (a 1‐point increase in novelty was associated with a 0.05‐point decrease in pain intensity, *p* < 0.001). There were no significant effects for unpredictability or social evaluative threat on overall pain intensity.

#### 3.2.2 Effects of Stress on BS Pain Intensity Variance (Scale)

Results showed that there was less BS variance (i.e., more homogeneity) in pain intensity ratings when individuals reported higher levels of stress relative to their usual stress levels (WS variance ratio = 0.899, 95% confidence interval (CI): 0.833–0.971) and when they reported higher levels of perceived lack of control relative to their usual perceived control levels (WS variance ratio = 0.968, 95% CI: 0.948–0.989). This means that the BS variance in pain intensity decreases by 10% (so more homogeneity across participants) for every one‐point increase in stress intensity and by 3% for every one‐point increase in perceived lack of control compared to their own mean. There was also less BS variance in pain intensity among individuals reporting higher BS levels of novelty (beyond their stress intensity); individuals reporting more novelty were more homogeneous in their pain scores than those reporting less novelty (BS variance ratio = 0.784; 95% CI: 0.665–0.924).

#### 3.2.3 Effects of Stress on WS Pain Intensity Variance (Scale)

There were no significant differences in WS variance in pain intensity based on stress levels or STUN characteristics. Individuals' pain ratings were not more consistent or erratic as a function of their stress levels.

### 3.3 Sensitivity Analyses

#### 3.3.1 Inclusion of All Participants With at Least One Diary Entry (*N* = 217)

The MELS model was run again using the entire sample. This analysis showed that the significance of the results remains unchanged.

#### 3.3.2 Inclusion of Participants With at Least 10 Diary Entries and Including All Stress Intensity Scores Including 0

For the mean model, there was no longer a significant association between BS control with pain intensity; however, there was a significant association between BS social evaluative threat (ego) and pain intensity. In the BS model, WS stress intensity, WS control and BS novelty were no longer associated with BS variance in pain ratings. There was a significant random location (mean) effect of WS variance, meaning that participants with higher average pain intensity are less consistent in their ratings, and participants with lower average pain intensity are more consistent in their ratings. Those results are presented in File S3.

#### 3.3.3 Inclusion of Participants With at Least 10 Diary Entries and Who Have at Least Seven Diaries With Stress Intensity Score > 0

A total of 157 participants out of the 181 had at least seven diary entries with a stress intensity score > 0. The MELS model was run with these participants (see File S4). Results showed no differences with the model obtained in the main analysis for the stress intensity. However, in the mean model, BS control was no longer significantly associated with pain intensity. In the BS model, WS control and BS novelty were no longer associated with BS variance in pain ratings.

#### 3.3.4 Inclusion of Participants With at Least 10 Diary Entries and Who Have at Least Seven Diaries With Stress Intensity Score ≥ 4

A total of 71 participants out of the 181 had at least seven diary entries with a stress intensity score ≥ 4 and the MELS model was ran with this subsample (see File S5). In the mean model, WS novelty was no longer associated with pain intensity. In the BS model, WS control was no longer associated with BS variance in pain ratings. The random location (mean) effect on WS pain variance was significant, meaning that participants with higher average pain intensity are less consistent in their ratings, and participants with lower average pain intensity are more consistent in their ratings.

## 4 Discussion

The associations between STUN characteristics, stress, and pain intensity in individuals living with cLBP were studied using ecological momentary assessments. When examined simultaneously, two STUN characteristics were significantly associated with current pain intensity beyond the overall effect of stress, although effect sizes were relatively small. A greater perceived lack of control over a situation was associated with higher pain intensity, while higher levels of WS novelty were associated with lower pain intensity. Social‐evaluative threat and unpredictability showed no significant associations with pain intensity. There was more homogeneity in pain scores across participants at higher levels of within‐participant stress intensity, within‐participant perceived lack of control, and between‐participant perceived novelty. Participants' own pain intensity ratings (WS variance) were not more or less consistent or erratic as a function of their stress levels.

### 4.1 Stress Components Associated With Pain Intensity Scores

Higher levels of stress intensity between participants and within participants were associated with higher pain intensity levels. These associations were consistent across all models run in the sensitivity analyses. These findings suggest that individuals with overall higher levels of stress report higher levels of pain intensity. They align with existing evidence suggesting that prolonged stress exposure exacerbates pain by contributing to the dysregulation in stress‐response systems such as the HPA axis (Borsook et al. 2012; Vachon‐Presseau et al. 2013).

Results also showed that when individuals experience higher stress levels than their own mean, pain intensity also increases. As such, regardless of whether an individual has an overall low or high level of stress, a temporary increase in stress intensity increases pain levels. The literature is scarce on the association between stress variations and chronic pain. One study has shown that individuals who self‐perceive having higher levels of stress reactivity were more likely to develop chronic pain conditions compared to those reporting lower levels of stress reactivity (Boring et al. 2023). Overall, these findings underscore the importance of considering stress and pain as dynamic and interrelated experiences (Stone, Obbarius, et al. 2021).

Beyond stress intensity levels, two STUN characteristics, sense of low control and novelty, were associated with pain intensity. Results were inconsistent across the sensitivity analyses, but the most replicated findings were the associations between WS perceived lack of control and novelty with pain intensity scores. Regardless of whether an individual is typically reporting overall high or low levels of perceived control or novelty compared to others, experiencing momentary increases in their own levels of perceived lack of control or momentary decreases in perceived novelty was associated with increased pain intensity. Effects sizes were small to very small however, indicating that stress intensity is probably having a larger impact on pain intensity than the STUN components.

In the broader literature, perceived lack of control is often, but not always associated with increased pain intensity (see (Ghoussoub et al. 2024) for a review), but those studies measured overall perceived control over pain and not within‐individual fluctuations in levels of perceived control. Experimental manipulations of the level of control over painful stimuli pain intensity show mixed results (Ghoussoub et al. 2024). Even less literature exists for the concept of novelty of stressors in chronic pain or general populations and typically results are inconsistent across studies (Defrin et al. 2008; Ronga et al. 2013; Slepian et al. 2017). Similar constructs, however, have been widely studied related to pain itself, such as habituation (adaptive adjustments to repetitive or continuous pain stimulation that leads to decreased perceived pain intensity) and sensitization (increased pain perception resulting from repeated exposure to a painful stimulus). There is evidence that individuals with chronic pain might have defective habituation processes to events that are painful, particularly when there is a perception of potential risk of harm (Harrison et al. 2025; van der Miesen et al. 2024). When examining the role of novelty of stressful situations unrelated to pain, the literature suggests that novel experiences may divert attention and increase engagement with external environments, thereby reducing pain perception (McCaul and Malott 1984). However, novelty ratings of a stressor inevitably decrease over time. The results suggest that new sources of stress may temporarily distract from pain, but it is unclear whether this effect is sustained.

Unpredictability and social evaluative threats were not significantly associated at the BS or WS level with pain intensity scores. This might be because the overall level of stress intensity accounts for most of the stress‐related variance in pain intensity scores (and this might be also true for control and novelty given the small effect sizes). It is also possible that interactions between STUN characteristics may further complicate these relationships; perceiving a lack of control might be more stressful (and likely impact pain intensity) when it is predictable (and thus unavoidable). No other studies have used a similar methodology to measure these stress components in real time in a clinical population.

### 4.2 Stress Components Associated With Pain Variability Across Participants

Increased WS stress levels were associated with reduced heterogeneity in pain intensity ratings across participants, and this result was also found in most of the sensitivity analyses. This suggests higher stress levels may contribute to more uniform pain experiences across participants. In other words, at lower levels of stress compared to their own mean, individuals are more heterogeneous in their pain scores, but when they experience higher stress levels than it is typical for them, they are more similar in their pain ratings. This might indicate that when an individual is experiencing lower stress levels than their own mean, pain intensity ratings are more variable and likely influenced by a multitude of other factors. However, an increase in stress intensity might more consistently impact pain experiences across participants.

These findings align with adaptation‐level theory, which suggests that pain perception is influenced by an internal reference point shaped by experiences. Frequently being exposed to stress may elevate this adaptation level, contributing to stress‐induced hyperalgesia, where even mild pain is perceived as more intense (Rollman 1979). Stress‐induced hyperalgesia represents maladaptive neurobiological changes in pain processing pathways that take place with exposure to repeated or chronic stress and is frequent in individuals with chronic pain conditions (Jennings et al. 2014; Loffler et al. 2023; Olango and Finn 2014). It is thus possible that individuals who report increased stress compared to their own baseline are more consistent in their pain ratings (e.g., when stress levels increase pain ratings are more consistently high).

No STUN characteristic was systematically associated with BS variance in pain ratings across the main analyses and the sensitivity analyses, suggesting again that perhaps stress intensity levels are more strongly associated with pain intensity ratings than the perceived individual characteristics of stressors.

### 4.3 Stress Components Associated With Pain Variability Within Participants

No stress intensity or STUN characteristics were associated with WS variance in pain ratings, suggesting that participants are not more or less consistent/erratic in their own pain intensity ratings as a function of stress levels. In other words, the degree to which individuals are consistent or not in their pain ratings is not a function of stress levels. This is likely a reflection of the complex and multidimensional nature of the pain experience; many internal and external factors might influence variations in pain scores beyond stress levels.

### 4.4 Clinical Implications and Future Directions

Results of this study mostly show that participants with higher levels of stress, but also individuals, regardless of their own mean stress intensity, experiencing heightened stress intensity are reporting higher pain intensity levels. This implies that stress management interventions in the context of chronic pain should not only be offered to those presenting with overall high levels of stress. Individuals with mild to moderate levels of stress might benefit from learning strategies to manage transient increases in stress as to minimise their impact on pain perceptions.

Future research should clarify the role of STUN characteristics, above stress intensity, in pain intensity and pain variability. Perceived lack of control and novelty might be interesting potential targets of interventions; however, these results should be confirmed in other studies. The present study was limited by the use of time‐based measurement and unvalidated STUN measures. It will be important to continue exploring how STUN characteristics are associated with pain‐related and non‐pain‐related stressors, and during event‐based intensive stress measurement. The interacting effects of STUN characteristics (e.g., uncontrollable stressful situation that is predictable) on pain intensity and pain variability should also be explored.

Existing therapies like cognitive behavioural therapies, acceptance and commitment therapies, and pain reprocessing therapies often focus on global stress appraisal and acceptance but do not explicitly incorporate STUN characteristics. Perhaps interventions that help individuals recognise when low control exacerbates pain and apply tailored coping strategies could help further manage pain states. The use of real time assessments of perceived low control may offer insights into how individuals perceive their environment in relation to their pain, which could be explored in future interventional research. Novelty‐based interventions could complement existing approaches by promoting engagement in new, non‐threatening experiences to reduce pain perception.

The WS findings suggest that momentary increases in stress are associated with higher pain intensity, highlighting a potential area for future research on the role of in‐the‐moment stress management in pain modulation. Incorporating momentary assessments of perceived control into research and clinical settings may help identify contexts in which low perceived control coincides with higher pain levels. Such insights could eventually contribute to the development of more tailored coping strategies, although further research is needed to establish their clinical relevance.

### 4.5 Strengths and Limitations

Ecological momentary assessments allowed to study the dynamic nature of stress and pain. Multiple recruitment methods were employed, and the study achieved a high completion rate (83%). Unlike most observational studies focusing on global stress, this study examined STUN characteristics (Ghoussoub et al. 2024). However, there are limitations.

The sample lacked diversity, with most participants identifying as White and none identifying outside the male/female binary, limiting generalizability. Future research should target equity‐deserving populations with cLBP.

A quarter of eligible individuals declined participation, possibly due to lack of required technology or concurrent enrollment in the larger online study from which they were recruited. Efforts were made to minimise selection bias by providing iPods to those without smartphones or tablets, and a team member ensured they could use it.

This study adopted a fixed schedule for diaries to match saliva sampling (part of the larger study). This decision limits the generalizability of the findings, and future studies should consider an event‐based design or the use of random prompts.

Diary questions used numerical responses for ease and higher completion rates, but these limited capturing nuanced experiences (Pagé et al. 2021a). The STUN questions were developed for the purpose of this study and results are thus exploratory in nature. Future studies should examine the psychometric properties of these variables. The lack of validated psychometric properties for single‐item questions introduces interpretation variability, despite thorough explanations of the STUN model. In addition, the diary measured pain intensity only and pain interference was not captured. While the goal of the manuscript was to examine associations between pain intensity and stress, the absence of a pain interference measure limits the transferability of the findings to clinical contexts and theoretical interpretations within broader biopsychosocial models of pain. Similarly, many other variables, including mood, sleep, work, social interactions, physical activity levels and medications could influence momentary pain intensity scores, and future work should explore how these factors might interact with stress variables in their association with pain intensity.

The analyses excluded diary entries where the stress intensity was 0 (664 observations out of 3077), to be able to test the effects of the STUN characteristics. However, this decision limits the generalizability of the results to all daily situations (stressful or not). Future research could use statistical models that include all levels of stress intensity.

STUN characteristics were examined in a cLBP sample. Future studies should extend this research to other pain populations to assess the STUN model's broader relevance.

## 5 Conclusion

Stress intensity is associated with pain intensity and to between‐participant variance in pain ratings. STUN characteristics were inconsistently associated with pain variance, but lack of perceived control and novelty might be associated with pain intensity. Incorporating real time assessments into clinical practice may help develop more tailored, effective pain management strategies that account for perceived situational stressors and their impact on pain intensity.

## Author Contributions

This study was designed by M.G.P., P.R., S.L, L.D., M.R. and É.V.‐P. The data collection was performed by K.G., M.G.‐M., É.D. and M.G.P. The data was analysed by K.G. and M.G.P., and the results were critically examined by all authors. K.G. had a primary role in preparing the manuscript, which was edited by M.G.P. All authors have approved the final version of the manuscript and agree to be accountable for all aspects of the work.

## Funding

Canadian Institutes of Health Research, grant number 436406. MG Pagé is a Junior 2 research scholar from the Fonds de recherche du Québec‐Santé (FRQS). K Ghoussoub is the recipient of a doctoral scholarship of the Université de Montréal (bourse de la montagne).

## Disclosure

Generative artificial intelligence (AI) was not used in the preparation of this manuscript.

## Conflicts of Interest

The authors declare no conflicts of interest.

## Supporting information


**Data S1:** ejp70240‐sup‐0001‐SupplementaryFiles.pdf.

## Data Availability

The data can be made available upon reasonable request and pending approval from our research ethics board.
