# Supplementary material for: Stress Characteristics and Their Associations With Pain Intensity: An Ecological Momentary Assessment Study
Source: Eur J Pain. 2026 Feb 25;30(3):e70240. doi: 10.1002/ejp.70240 (PMC12936249; doi:10.1002/ejp.70240)
Supplement: Supplementary file 1 — Data S1: ejp70240‐sup‐0001‐SupplementaryFiles.pdf. [file EJP-30-0-s001.pdf]

Supplementary File 1. Questionnaires administered – Stress-pain study

Questionnaire	*Baseline*	*Diary*	*Follow-up*
S.T.U.N questionnaire	X		
NIH minimal dataset[1,2]	X		X
Perceived Stress Scale[3]	X		
COVID Stress Scale[4]	X		
Pain Appraisal Inventory[5]	X		X
Short-form (4-item) Pain Catastrophizing Scale[6]	X		
4-item Short-Form of the Pain Self-Efficacy Questionnaire[7]	X		X
Gender Role Expectations of Pain Questionnaire[8]	X		
Stress-pain diary		X	
Oswestry Low Back Pain Disability Questionnaire[9]			X

References

1. Deyo RA, Dworkin SF, Amtmann D, et al. Report of the NIH Task Force on research standards for chronic low back pain. *Phys Ther.* 2015;95(2):e1-e18.
2. Lacasse A, Roy JS, Parent AJ, et al. The Canadian minimum dataset for chronic low back pain research: a cross-cultural adaptation of the National Institutes of Health Task Force Research Standards. *CMAJ Open.* 2017;5(1):E237-e248.
3. Cohen S, Kamarck T, Mermelstein R. A global measure of perceived stress. *Journal of Health and Social Behavior.* 1983;24:385-396.
4. Taylor S, Landry CA, Paluszek MM, Fergus TA, McKay D, Asmundson GJG. Development and initial validation of the COVID Stress Scales. *J Anxiety Disord.* 2020;72:102232.
5. Unruh AM, Ritchie JA. Development of the Pain Appraisal Inventory: Psychometric properties. *Pain Res Manag.* 1998;3(2):105-110.
6. Walton DM, Mehta S, Seo W, MacDermid JC. Creation and validation of the 4-item BriefPCS-chronic through methodological triangulation. *Health Qual Life Outcomes.* 2020;18(1):124.
7. McWilliams LA, Kowal J, Wilson KG. Development and evaluation of short forms of the Pain Catastrophizing Scale and the Pain Self-efficacy Questionnaire. *Eur J Pain.* 2015;19(9):1342-1349.
8. Robinson ME, Riley JL, 3rd, Myers CD, et al. Gender role expectations of pain: relationship to sex differences in pain. *J Pain.* 2001;2(5):251-257.
9. Fairbank JC, Pynsent PB. The Oswestry Disability Index. *Spine (Phila Pa 1976).* 2000;25(22):2940-2952; discussion 2952.

Supplementary File 2. Stress-pain diary

1) How much **pain** do you feel **right now**?
   0 = No pain at all
   10 = Worst pain imaginable

☐	☐	☐	☐	☐	☐	☐	☐	☐	☐	☐
0	1	2	3	4	5	6	7	8	9	10

2) How is your **mood** right now?
   0 = Not at all depressed
   10 = Most depressed I've ever felt

☐	☐	☐	☐	☐	☐	☐	☐	☐	☐	☐
0	1	2	3	4	5	6	7	8	9	10

3) How much **fatigue** do you feel right now?
   0 = No fatigue
   10 = Worst fatigue imaginable

☐	☐	☐	☐	☐	☐	☐	☐	☐	☐	☐
0	1	2	3	4	5	6	7	8	9	10

4) How **stressed** do you feel right now?
   0 = Not stressed at all
   10 = Most stressed imaginable

☐	☐	☐	☐	☐	☐	☐	☐	☐	☐	☐
0	1	2	3	4	5	6	7	8	9	10

5) How much of this stress is due to your **pain**?

0% due to my pain 100% due to my pain

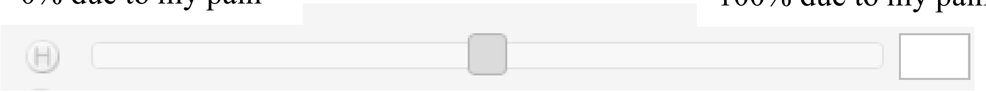

6) How much of this stress is due to the **pandemic**?

0% due to the pandemic 100% due to the pandemic

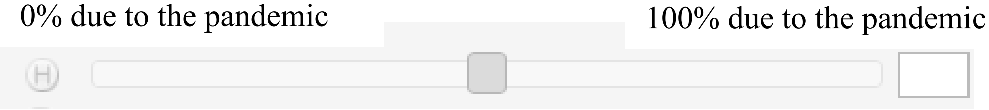

7) How much of this stress is due to other **things**?

0% due to other things — 100% due to other things

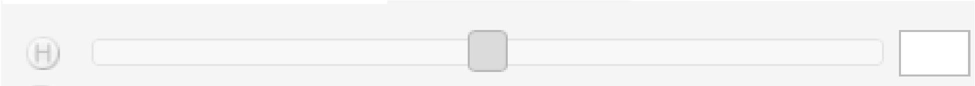

8) How much **control** do you feel you have over the stressful situation(s) you are currently facing

0% no control at all — 100% completely in

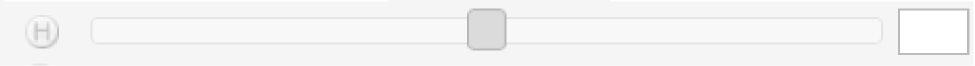

9) How **novel** (new) are the stressful situation(s) you are currently facing

0% not novel at all — 100% completely novel

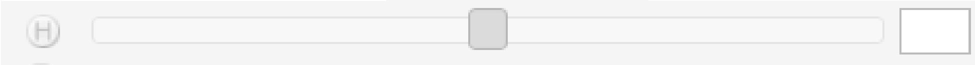

10) How **predictable/unpredictable** are the stressful situation(s) you are currently facing [0% = completely predictable, 100% = completely unpredictable]

0% completely predictable — 100% completely

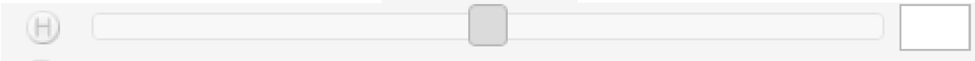

11) How **threatening to your personality** are the stressful situation(s) you are currently facing [0% = not threatening at all, 100% = extremely threatening]

0% not threatening at all — 100% extremely

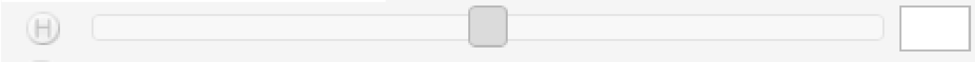

Supplementary File 3. Mixed-effects location scale models examining the associations between stress characteristics and pain intensity including participants with 10 or more diary entries and with all stress intensity scores including 0

Variable and Parameters	Estimate	*SE*	*p*	Ratio	95% CI	
					Lower	Upper
**Mean model**						
Intercept	3.203	0.305	< 0.001**	.	.	.
Stress (BS)	0.260	0.078	< 0.001**	.	.	.
Stress (WS)	0.365	0.045	< 0.001**	.	.	.
Control (BS) §	0.080	0.043	0.062	.	.	.
Control (WS)	0.062	0.022	0.004*	.	.	.
Novelty (BS)	-0.093	0.060	0.125	.	.	.
Novelty (WS)	-0.060	0.024	0.013*	.	.	.
Unpredictability (BS)	-0.110	0.062	0.076	.	.	.
Unpredictability (WS)	-0.005	0.021	0.801	.	.	.
Threat to ego (BS) §§	0.154	0.071	0.031*	.	.	.
Threat to ego (WS)	-0.022	0.032	0.502	.	.	.
**Between-subject variance in pain ratings**						
Intercept	0.664	0.376	0.078	1.942	0.929	4.060
Stress (BS)	-0.162	0.117	0.165	0.850	0.676	1.069
Stress (WS) §	-0.142	0.120	0.235	0.867	0.686	1.097
Control (BS)	-0.015	0.054	0.783	0.985	0.886	1.095
Control (WS) §	-0.017	0.042	0.689	0.983	0.905	1.068
Novelty (BS) §	-0.042	0.094	0.657	0.959	0.797	1.153
Novelty (WS)	-0.056	0.055	0.310	0.946	0.849	1.053
Unpredictability (BS)	0.120	0.073	0.101	1.127	0.977	1.301
Unpredictability (WS)	-0.021	0.035	0.544	0.979	0.915	1.048
Threat to ego (BS)	-0.042	0.095	0.659	0.959	0.795	1.156
Threat to ego (WS)	0.066	0.070	0.343	1.068	0.932	1.224
**Within-subject variance in pain ratings**						
Intercept	0.900	0.146	< 0.001**	2.459	1.849	3.271
Stress (BS) §§	0.111	0.037	0.002*	1.118	1.039	1.202
Stress (WS)	0.065	0.041	0.114	1.067	0.984	1.156
Control (BS)	-0.040	0.024	0.097	0.961	0.916	1.007
Control (WS)	-0.017	0.026	0.529	0.984	0.934	1.036
Novelty (BS)	-0.049	0.044	0.269	0.952	0.873	1.038
Novelty (WS)	0.027	0.027	0.332	1.027	0.973	1.084
Unpredictability (BS)	-0.043	0.036	0.237	0.958	0.892	1.029
Unpredictability (WS)	-0.006	0.025	0.823	0.995	0.948	1.044
Threat to ego (BS)	-0.028	0.035	0.433	0.973	0.908	1.042
Threat to ego (WS)	-0.033	0.031	0.286	0.968	0.911	1.028
**Random scale standard deviation**						
Standard Deviation §	0.001	0.043	0.999	1.245	1.060	1.463
**Random location (mean) effect on WS variance**						
Location Effect §§	0.219	0.082	0.008*	1.000	0.920	1.087

Note. *p < 0.05 **p < 0.001 § Variable that is no longer significant in this sensitivity analysis compared to the main analysis. §§ Variable that is now significant in this sensitivity analysis compared to the main analysis. BS: Between-subject; WS: Within-subject

Supplementary File 4. Mixed-effects location scale models examining the associations between stress characteristics and pain intensity carried out on participants with at least 10 diary entries and who have at least 7 diaries with stress intensity score ≥ 0

Variable and Parameters	Estimate	*SE*	*p*	Ratio	95% CI	
					Lower	Upper
**Mean model**						
Intercept	2.060	0.399	< 0.001**	.	.	.
Stress (BS)	0.565	0.078	< 0.001**	.	.	.
Stress (WS)	0.235	0.019	< 0.001**	.	.	.
Control (BS) §	0.099	0.055	0.072	.	.	.
Control (WS)	0.050	0.012	< 0.001**	.	.	.
Novelty (BS)	0.082	0.069	0.235	.	.	.
Novelty (WS)	-0.054	0.012	< 0.001**	.	.	.
Unpredictability (BS)	-0.113	0.076	0.138	.	.	.
Unpredictability (WS)	-0.006	0.012	0.591	.	.	.
Threat to ego (BS)	0.064	0.054	0.239	.	.	.
Threat to ego (WS)	0.012	0.016	0.447	.	.	.
**Between-subject variance in pain ratings**						
Intercept	0.433	0.470	0.356	1.543	0.615	3.872
Stress (BS)	0.058	0.092	0.525	1.060	0.886	1.268
Stress (WS)	-0.110	0.032	0.001**	0.896	0.842	0.954
Control (BS)	-0.053	0.067	0.420	0.948	0.833	1.079
Control (WS) §	-0.034	0.020	0.084	0.966	0.930	1.005
Novelty (BS) §	-0.144	0.090	0.111	0.866	0.726	1.033
Novelty (WS)	0.001	0.021	0.945	1.001	0.961	1.043
Unpredictability (BS)	0.109	0.096	0.254	1.116	0.924	1.347
Unpredictability (WS)	-0.001	0.019	0.944	0.999	0.962	1.036
Threat to ego (BS)	0.011	0.071	0.871	1.012	0.881	1.162
Threat to ego (WS)	0.016	0.028	0.564	1.016	0.962	1.074
**Within-subject variance in pain ratings**						
Intercept	0.633	0.209	0.002*	1.884	1.250	2.837
Stress (BS)	0.051	0.043	0.238	1.052	0.967	1.145
Stress (WS)	0.027	0.022	0.208	1.028	0.985	1.072
Control (BS)	-0.027	0.029	0.352	0.973	0.919	1.030
Control (WS)	-0.016	0.015	0.270	0.984	0.956	1.013
Novelty (BS)	-0.007	0.039	0.850	0.993	0.920	1.070
Novelty (WS)	-0.008	0.015	0.565	0.992	0.963	1.021
Unpredictability (BS)	-0.027	0.043	0.537	0.974	0.895	1.060
Unpredictability (WS)	-0.004	0.014	0.758	0.996	0.969	1.023
Threat to ego (BS)	-0.010	0.028	0.737	0.991	0.937	1.047
Threat to ego (WS)	-0.018	0.019	0.362	0.983	0.946	1.020
**Random scale standard deviation**						
Standard Deviation	0.622	0.049	< 0.001**	1.061	0.935	1.205
**Random location (mean) effect on WS variance**						
Location Effect	0.059	0.065	0.359	1.863	1.693	2.049

Note. *p < 0.05 **p < 0.001 § Variable that is no longer significant in this sensitivity analysis compared to the main analysis. BS: Between-subject; WS: Within-subject

Supplementary File 5. Mixed-effects location scale models examining the associations between stress characteristics and pain intensity carried out on participants with at least 10 diary entries and who have at least 7 diaries with stress intensity score ≥ 4

Variable and Parameters	Estimate	*SE*	*p*	Ratio	95% CI	
					Lower	Upper
**Mean model**						
Intercept [§]	0.406	0.947	0.668	.	.	.
Stress (BS)	0.701	0.144	< 0.001**	.	.	.
Stress (WS)	0.245	0.028	< 0.001**	.	.	.
Control (BS)	0.226	0.089	0.011*	.	.	.
Control (WS)	0.046	0.023	0.039*	.	.	.
Novelty (BS)	0.130	0.096	0.175	.	.	.
Novelty (WS) [§]	-0.020	0.020	0.315	.	.	.
Unpredictability (BS)	-0.094	0.120	0.434	.	.	.
Unpredictability (WS)	-0.020	0.018	0.283	.	.	.
Threat to ego (BS)	0.014	0.059	0.818	.	.	.
Threat to ego (WS)	0.033	0.024	0.164	.	.	.
**Between-subject variance in pain ratings**						
Intercept	1.184	1.392	0.394	3.269	0.213	5.062
Stress (BS)	0.152	0.211	0.471	1.164	0.770	1.759
Stress (WS)	-0.091	0.043	0.034*	0.913	0.839	0.993
Control (BS)	-0.108	0.130	0.408	0.898	0.696	1.158
Control (WS) [§]	0.065	0.037	0.081	1.067	0.992	1.147
Novelty (BS)	-0.328	0.146	0.024*	0.720	0.542	0.958
Novelty (WS)	-0.017	0.033	0.603	0.983	0.922	1.049
Unpredictability (BS)	0.090	0.170	0.597	1.094	0.784	1.526
Unpredictability (WS)	0.022	0.030	0.448	1.023	0.965	1.085
Threat to ego (BS)	-0.057	0.106	0.590	0.944	0.767	1.163
Threat to ego (WS)	0.015	0.043	0.726	1.015	0.934	1.103
**Within-subject variance in pain ratings**						
Intercept	1.258	0.513	0.014*	3.518	1.286	9.623
Stress (BS)	0.007	0.078	0.932	1.007	0.865	1.172
Stress (WS)	-0.011	0.031	0.729	0.989	0.932	1.051
Control (BS)	-0.054	0.049	0.265	0.962	0.861	1.042
Control (WS)	-0.039	0.024	0.100	0.958	0.919	1.008
Novelty (BS)	-0.043	0.051	0.402	1.026	0.866	1.059
Novelty (WS)	0.026	0.024	0.293	0.953	0.978	1.076
Unpredictability (BS)	-0.048	0.070	0.495	0.960	0.831	1.094
Unpredictability (WS)	-0.040	0.021	0.057	0.960	0.921	1.001
Threat to ego (BS)	-0.016	0.038	0.684	0.985	0.914	1.061
Threat to ego (WS)	-0.008	0.028	0.763	0.992	0.939	1.047
**Random scale standard deviation**						
Standard Deviation	0.579	0.072	< 0.001**	0.756	0.624	0.915
**Random location (mean) effect on WS variance**						
Location Effect [§§]	0.045	0.097	0.004*	1.814	1.576	2.087

Note. *p < 0.05 **p < 0.001 [§] Variable that is no longer significant in this sensitivity analysis compared to the main analysis. [§§] Variable that is now significant in this sensitivity analysis compared to the main analysis. BS: Between-subject; WS: Within-subject
